# Non‐discontinuation of antiseizure medication in seizure‐free patients with epilepsy: Reasons and predictors among neurologists and patients

**DOI:** 10.1111/epi.18519

**Published:** 2025-06-26

**Authors:** Maria Ilyas‐Feldmann, Luise Graf, Jakob I. Doerrfuss, Matthias Dipper‐Wawra, Nicoletta Doerr, Rebekka Lehmann, Christian Meisel, David Steinbart, Mirja Steinbrenner, Martin Holtkamp

**Affiliations:** ^1^ Department of Neurology with Experimental Neurology Charité–Universitätsmedizin Berlin, corporate member of Freie Universität Berlin and Humboldt‐Universität Zu Berlin Berlin Germany; ^2^ Epilepsy Center Berlin‐Brandenburg Institute for Diagnostics of Epilepsy Berlin Germany

**Keywords:** drug withdrawal, outcome, pharmacotherapy, seizure freedom, seizure recurrence risk

## Abstract

**Objective:**

This study was undertaken to investigate clinical and psychosocial factors associated with antiseizure medication (ASM) non‐discontinuation in seizure‐free patients with epilepsy among both neurologists and patients.

**Methods:**

In this cross‐sectional study, neurologists documented their recommendations on ASM discontinuation (comprising both complete discontinuation and ≥25% dose reduction) in patients aged ≥18 years who had been seizure‐free for at least 2 years. Based on these recommendations, patients made individual decisions. In both neurologists and patients, reasons for and predictors of ASM non‐discontinuation were assessed considering demographic and epilepsy‐related variables as well as standardized psychosocial questionnaires.

**Results:**

Among 196 patients (53.1% female, median age = 50 years, interquartile range [IQR] = 36–61; median seizure‐free duration = 6 years, IQR = 4–11), neurologists recommended ASM discontinuation in 110 cases (56.1%), of which 29 patients (26.4%) agreed. Neurologists were more likely to favor ASM non‐discontinuation if they had fewer years of professional experience (odds ratio [OR] = .96, 95% confidence interval [CI] = .92–.99) and if patients had shorter seizure‐free durations (OR = .98, 95% CI = .98–.99). Among patients, longer seizure‐free duration (OR = 1.01, 95% CI = 1.01–1.02) and a history of generalized or focal to bilateral tonic–clonic seizures (OR = 2.72, 95% CI = 1.15–6.43) were independently associated with ASM non‐discontinuation. Excluding the 27 patients who favored a dose reduction, ASM non‐discontinuation was still associated with a longer duration of seizure freedom (OR = 1.02, 95% CI = 1.01–1.03). Further predictors were higher anxiety scores (OR = 1.37, 95% CI = 1.05–1.78) and better ASM tolerability (OR = 1.04, 95% CI = 1.01–1.07).

**Significance:**

Neurologists and, even more so, patients are hesitant to discontinue ASM, which was accomplished in only 15% of seizure‐free patients. Duration of seizure freedom has a major impact on the decision but in opposite directions comparing both groups. Understanding these differing perspectives is essential to improve shared decision‐making on this complex issue in epilepsy care.

**Key points**

- Among 196 seizure‐free patients, neurologists recommended ASM discontinuation in 110; of those, 29 patients agreed to discontinue.
- In neurologists, shorter clinical experience and shorter duration of patients' seizure freedom predicted ASM non‐discontinuation.
- Across all patients, ASM non‐discontinuation was associated with longer duration of seizure freedom and a history of generalized or bilateral tonic–clonic seizures.
- Excluding patients who only opted for dose reduction, ASM non‐discontinuation was additionally linked to higher anxiety and better ASM tolerability.
- Awareness of these influencing factors may support more effective shared decision‐making regarding ASM discontinuation in seizure‐free patients.

## 1 INTRODUCTION

In epilepsy, pharmacotherapy is the mainstay of treatment; approximately two out of three patients become seizure‐free with antiseizure medication (ASM).^1^ However, regular ASM intake may cause adverse events, which are reported spontaneously by 10%–40% of patients^2^; the rate rises to 60%–90% if structured questionnaires are used.^3^ Therefore, in patients with long‐term seizure freedom who are still on ASM, the question arises whether and when ASM can be discontinued. Guidelines recommend that ASM discontinuation may be considered after a minimum period of 2 years of seizure freedom.^4^, ^5^, ^6^ Discontinuation should be discussed with patients in a shared decision‐making process, weighing the benefit of stopping an adverse event‐prone medication against the risk of seizure recurrence.^7^ The latter would significantly impact psychosocial aspects, such as driving privileges and occupational issues; therefore, patients' corresponding fears and concerns need to be considered.

The risk of seizure recurrence within 2 years after discontinuation of ASM is approximately 40%–50%, which is twice as high as continuing ASM.^8^, ^9^ Following a meta‐analysis with more than 1700 patients, predictive variables for seizure recurrence after stopping ASM, such as a shorter seizure‐free interval until discontinuing ASM, have been identified.^10^ However, even if patients are suitable candidates for successful ASM discontinuation, only 26%–45% decide to do so.^11^, ^12^ A significant number of uncertainties regarding non‐discontinuation of ASM persist.^13^ These are reflected in the finding that discontinuation of ASM is discussed between physicians and seizure‐free patients in every third case,^11^ and it is realized in only every fifth subject.^12^, ^13^ Although previous studies have examined either physicians' or patients' perspectives on ASM discontinuation,^12^, ^13^, ^14^, ^15^, ^16^, ^17^ there remains a lack of integrated data on how these decisions unfold within the same clinical encounter. To our knowledge, no study to date has systematically assessed both neurologists' recommendations and patients' preferences in a seizure‐free patient population using a structured and comparative design. Moreover, no study has investigated whether specific personality traits, attitudes toward epilepsy or its treatment, or patient‐reported quality of life markers influence the decision to discontinue ASM.

In this cross‐sectional study, we aimed to gain deeper insight into the attitudes of neurologists and their seizure‐free patients toward ASM discontinuation. For both groups, we sought to identify reasons for and to analyze clinical and psychosocial variables independently associated with ASM non‐discontinuation.

## 2 MATERIALS AND METHODS

### 2.1 Study design

Consecutive patients who were at least 2 years seizure‐free and who were on ASM monotherapy were recruited between October 2022 and April 2024 from four epilepsy outpatient clinics in Berlin, Germany. These included three academic epilepsy outpatient clinics affiliated with the Department of Neurology, Charité–Universitätsmedizin Berlin, located at different sites across the city and operated independently by different neurologist teams, and one nonacademic epilepsy outpatient clinic at the Epilepsy Center, Evangelisches Krankenhaus Königin Elisabeth Herzberge, Berlin, Germany, at a fourth site in the city.

All patients received written study information prior to inclusion and signed informed consent forms. We excluded patients with moderate to severe intellectual disability, as documented in the medical record, if they were unable to independently participate in treatment decisions. Additionally, any patient under legal guardianship was excluded, as the local ethics committee does not allow inclusion of such patients. Patients with insufficient German language skills were also excluded, as multiple self‐administered questionnaires had to be completed.

The study was approved by the local ethics committee (EA4/129/22) and prospectively registered in the German Clinical Trials Register (ID: DRKS00030094).

**FIGURE 1:**
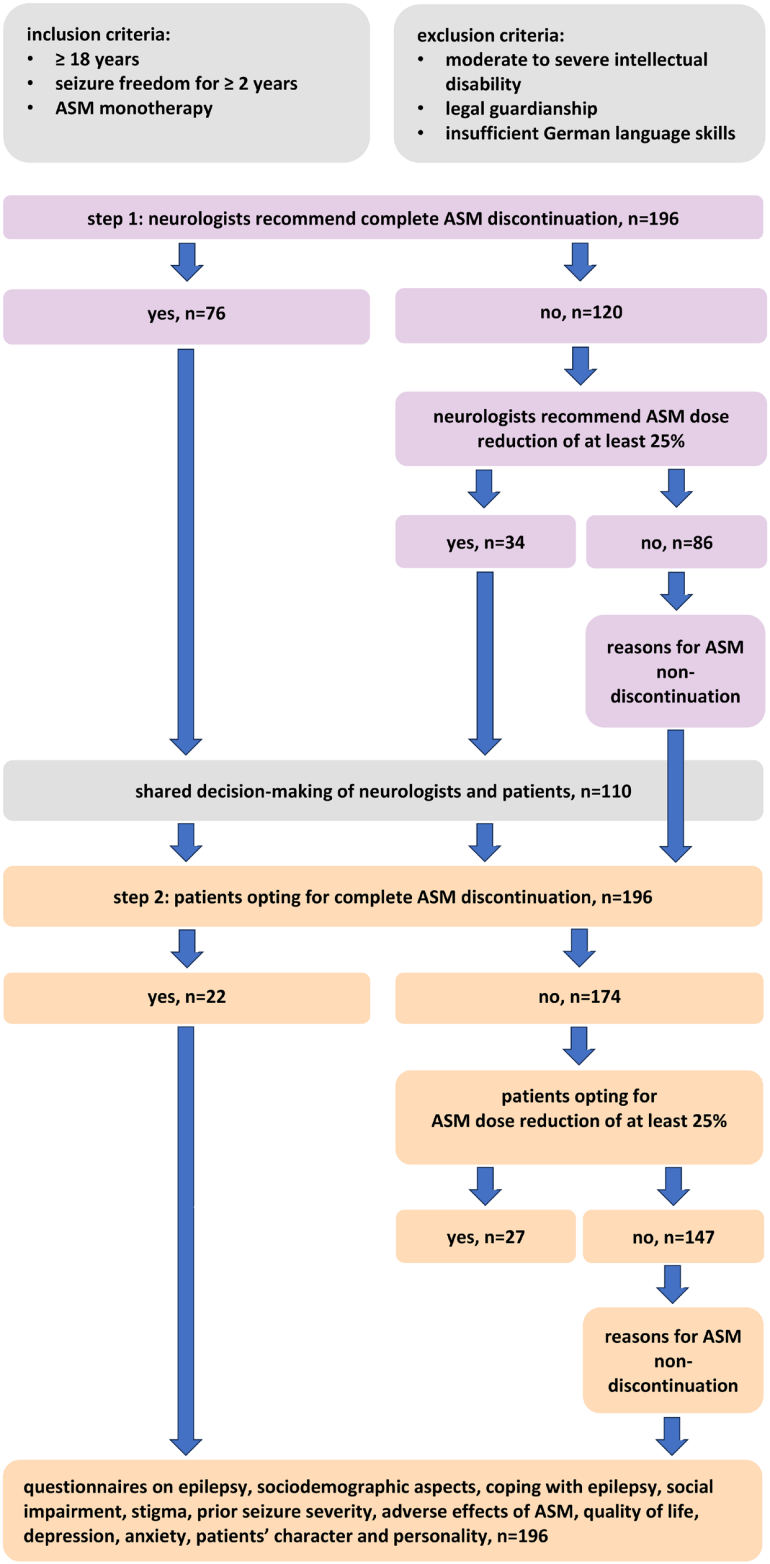
Stepwise study design and participant flow. Illustration shows the two‐step study design reflecting real‐world clinical decision‐making. First, neurologists assessed whether complete discontinuation of antiseizure medication (ASM) was appropriate. If not, they evaluated whether at least a ≥25% dose reduction was feasible. If the answer was yes for one of these two options, a shared decision‐making conversation with patients followed. If both options were declined, neurologists documented predefined or free‐text reasons for ASM non‐discontinuation. The same stepwise approach was applied to patients after their consultation. In addition, all patients completed standardized questionnaires on prior seizure severity, ASM adverse effects, quality of life, depression, anxiety, personality traits, and social context and stigma.

Our study followed a stepwise design to reflect real‐world clinical practice (Figure 1). First, neurologists assessed seizure‐free patients to determine whether they would recommend complete ASM discontinuation. This assessment was based solely on the neurologists' clinical judgment and occurred before the patient consultation; it did not represent a final treatment recommendation or even decision but rather a theoretical appraisal of suitability, assuming the patient would be open to discontinuation. If this was not deemed appropriate, they were then asked whether they would at least consider a significant dose reduction of 25%, explicitly as a first step toward eventual discontinuation. This approach acknowledges that ASM discontinuation is often not an all‐or‐nothing decision, but rather a gradual process. For consistency in terminology, and because both scenarios reflect the neurologists' intent to completely discontinue or to significantly reduce the dose of the ASM, we refer to both complete ASM discontinuation and dose reduction as “discontinuation” throughout the article. The term “non‐discontinuation” was deliberately chosen to reflect an active decision of neurologists and patients not to pursue any form of ASM reduction, distinguishing it from passive or default “continuation.” If neurologists considered a seizure‐free patient suitable for discontinuation, they discussed this option with the patient in a shared decision‐making process. For patients choosing discontinuation, the ASM dose was gradually reduced over several weeks, depending on the specific ASM. Complete discontinuation or a dose reduction of at least 25% was expected to be achieved within 6 months, that is, before the patient's next outpatient clinic visit. If neurologists did not recommend ASM discontinuation, they had to document their reasoning in the patient's medical records. For standardization, we established a predefined list of reasons based on clinical variables that have been independently associated with seizure recurrence after ASM discontinuation, as identified in a previously published meta‐analysis.^10^ This standardization was implemented to ensure consistent documentation across study sites and to allow structured comparison of decision‐making patterns. Importantly, because neurologists first decided whether to recommend ASM discontinuation and only subsequently documented their reasons in cases of non‐discontinuation, the standardized list did not influence or guide clinical decisions. The reasons comprised the following: history of febrile seizures, short seizure‐free period, age at seizure onset > 25 years, ≥1 prior ASM discontinuation attempt(s), ≥10 seizures before reaching seizure freedom, and long duration of epilepsy before remission; a free text field for giving any additional reasons was provided. Additionally, we collected data on the neurologists' age, sex, and years of clinical experience in neurology.

In the second step, all patients completed electronic questionnaires—either on‐site or online from home—regardless of whether their neurologists had advocated for ASM discontinuation (Figure 1). One of the authors (L.G.) assisted patients in understanding the questions, either face‐to‐face or by telephone. Importantly, the questionnaires were completed after the consultation with the neurologist. In patients for whom ASM discontinuation was not recommended by the neurologists, the topic was often not actively discussed, so responses likely reflect their independent perspective. In contrast, where discontinuation was recommended, a shared decision‐making conversation typically preceded questionnaire completion. Patients were first asked the same question as their neurologists: whether they would opt to completely discontinue their ASM. If they did not favor this option, they were then asked whether they would consider a dose reduction of at least 25% as an initial step toward eventual ASM discontinuation. For consistency, as with neurologists, we refer to both complete ASM discontinuation and dose reduction as “discontinuation,” and patients who chose neither are referred to as opting for “non‐discontinuation.” If patients opted for ASM non‐discontinuation, they were asked to choose one or more reasons from a predefined list that was based on a previous study on why seizure‐free patients decide against stopping ASM.^12^ This list comprised the following: feeling safe and well‐adjusted to ASM, fear of seizure recurrence, fear of temporarily losing driving privileges, many and/or severe seizure(s) in past, seizure recurrence(s) after previous discontinuation attempt(s), and fear of losing work; furthermore, a free text field for any additional reasons was provided. In addition, patients completed validated structured questionnaires including the German Performance, Socio‐Demographic Aspects, Subjective Evaluation questionnaire, which collects information on the patients' social environment, coping with epilepsy, social impairment, and perceived stigma.^18^ Furthermore, we implemented German translations of questionnaires assessing prior seizure severity (using the Liverpool Seizure Severity Scale),^19^ adverse effects of ASM (Liverpool Adverse Events Profile),^20^ quality of life (Patient‐Weighted Quality of Life in Epilepsy [QOLIE‐31‐P]),^21^ depression (Neurological Disorders Depression Inventory for Epilepsy),^22^ anxiety (Generalized Anxiety Disorder–7 items [GAD‐7]),^23^ and patients' character and personality (Big Five Inventory, a 10‐item version).^24^ We also collected patients' demographic data and extracted further information from their medical records regarding seizure type(s), epilepsy type and syndrome, etiology, total number of previous seizures (1–9 seizures vs. ≥10 seizures), duration of epilepsy before remission, duration of seizure freedom, and current ASM and dosage as well as total number of ASMs (including current).

For each patient, the ASM load was calculated as ratio according to the 2020 World Health Organization Center for Drug Statistics Methodology ATC/DDD Index using the formula “individual ASM dosage per day divided by defined daily dose.”

Considering all available demographic and clinical data of patients, we retrospectively calculated 2‐ and 5‐year seizure recurrence risks after possible ASM discontinuation according to the online available “antiepileptic drug withdrawal risk calculator score” (http://epilepsypredictiontools.info/aedwithdrawal, UMC Utrecht, the Netherlands^10^). The scores were calculated using a Microsoft Excel script provided to us by the calculator's authors. As seizure relapse risk calculators are not part of our clinical routine, neither neurologists' recommendations nor patients' preferences were directly influenced by such tools.

Patients who chose to discontinue ASM were followed up 6 months after inclusion to assess whether they realized complete discontinuation or dose reduction and to ask for potential seizure recurrence.

### 2.2 Statistical analysis

Study data were collected and managed using the REDCap (Research Electronic Data Capture) tools hosted at Charité–Universitätsmedizin Berlin.^25^ All statistical analyses were performed using IBM SPSS Statistics 30.0. Nominal and ordinal variables were reported as frequencies and percentages. Univariable analyses for categorical variables were performed using Pearson chi‐squared test. Metric variables were tested for normality with the Kolmogorov–Smirnov test. Nonnormally distributed variables are reported as median (interquartile range [IQR]) and analyzed with the Mann–Whitney *U*‐test, whereas normally distributed variables are reported as mean (SD) and were analyzed using the independent samples *t*‐test. All tests were two‐sided, with *p* < .05 considered statistically significant. Adjusted multiple logistic regression analysis (enter method) was conducted to calculate odds ratios (ORs) with 95% confidence intervals (CIs) for factors independently associated with ASM non‐discontinuation favored by both neurologists and patients. Variables that were statistically significant in univariable analysis and those deemed clinically relevant were included. To avoid multicollinearity, the calculated 2‐ and 5‐year recurrence risks were excluded from the model.

## 3 RESULTS

### 3.1 Study population

#### 3.1.1 Neurologists

Overall, 10 neurologists (four female; median age = 39 years, IQR = 33–45 years) consulted their patients on possible ASM discontinuation. Median duration of experience in neurology was 12 years (IQR = 6–18 years).

#### 3.1.2 Patients

**FIGURE 2:**
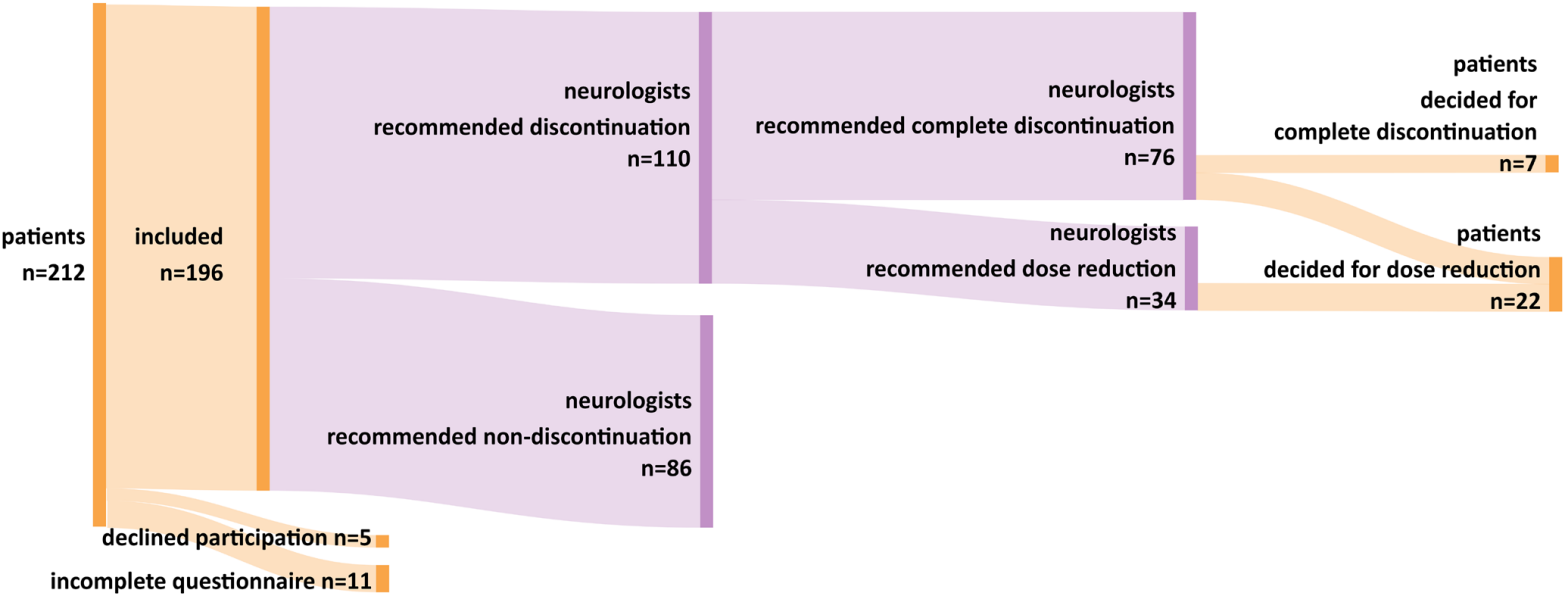
Decision pathways of antiseizure medication (ASM) discontinuation. Pathways of study participants based on neurologists' recommendations and patients' decisions in the shared decision‐making process regarding ASM discontinuation are shown. Eleven of 76 patients for whom the neurologists recommended complete discontinuation of ASM decided to reduce the ASM dose instead. Note that <10% (*n* = 7) of these 76 patients consented to completely stop the ASM; this is <4% of the entire study population.

In the 18‐month recruiting period, a total of 212 patients fulfilled the inclusion criteria and were eligible to participate. Five patients declined participation, and 11 patients did not entirely complete the questionnaires. Finally, data for 196 patients were analyzed (Figures 1 and 2). Of those, 90 patients (45.9%) completed the questionnaires on‐site and 106 patients (54.1%) online at home. There were no significant differences between the groups regarding age, duration of epilepsy before remission, and duration of seizure freedom. However, in the group that completed the questionnaires on‐site, there were significantly more females (61.1% vs. 46.2%, *p* = .037), and patients significantly more often opted for discontinuation of ASM (32.2% vs. 18.9%, *p* = .031).

The median age of the analyzed 196 patients was 50 years (IQR = 36–61 years), and 53.1% were female. Median duration of epilepsy before remission was 9 years (IQR = 1–20 years), and median duration of terminal seizure freedom was 6 years (IQR = 4–11 years; Table 1).

**TABLE 1:** Clinical variables associated with recommendation by neurologists of ASM non‐discontinuation.

Variable	Total, *n* = 196	Non‐discontinuation, *n* = 86	Discontinuation, *n* = 110	Univariable analysis	Logistic regression analysis, OR (95% CI)
Neurologists^a^					
Female sex, *n* (%)	68 (34.7)	31 (36.1)	37 (33.6)	*p* = .725^b^	Not included
Age, years, median (IQR)	39 (38–52)	38 (34–52)	41 (38–55)	***p* = .002** ^c^	Not included^d^
Duration of experience as neurologists, years, median (IQR)	14 (11–25)	11 (7–25)	15 (11–27)	***p* = .002** ^c^	.**96 (.92–.99)**
Patients					
Female sex, *n* (%)	104 (53.1)	41 (47.7)	63 (57.3)	*p* = .181^b^	Not included
Age, years, median (IQR)	50 (36–61)	46 (34–65)	52 (38–61)	*p* = .284^c^	Not included^e^
Relationship status “single”, *n* (%)	51 (26.0)	30 (34.9)	21 (19.1)	***p* = .012** ^b^	2.01 (.93–4.33)^f^
Age at epilepsy onset, years, median (IQR)	21 (14–42)	23 (16–47)	20 (13–37)	*p* = .115^c^	1.01 (.99–1.03)
Duration of epilepsy until remission, years, median (IQR)	9 (1–20)	10 (2–19)	9 (1–21)	*p* = .809^c^	1.01 (.98–1.04)
Duration of seizure freedom, years, median (IQR)	6 (4–11)	5 (3–7)	9 (5–14)	***p* < .001** ^c^	.**98 (.98–.99)**
Previous generalized or focal to bilateral tonic–clonic seizures, *n* (%)	163 (83.2)	74 (86.1)	89 (80.9)	*p* = .340^b^	Not included
Total number of seizures ≥ 10, *n* (%)	63 (32.1)	28 (32.6)	35 (31.8)	*p* = .912^b^	1.22 (.55–2.70)^f^
Epilepsy type, *n* (%)					
Focal	119 (60.7)	54 (62.8)	65 (59.1)		
Generalized	48 (24.5)	22 (25.6)	26 (23.6)	*p* = .543^b^	Not included
Unknown	29 (14.8)	10 (11.6)	19 (17.3)		
Structural epilepsy etiology, *n* (%)	52 (26.5)	30 (34.9)	22 (20.0)	***p* = .019** ^b^	1.40 (.65–3.04)^f^
Total number of ASMs [including current ASM], median (IQR)	2 (1–3)	1 (1–3)	2 (1–3)	*p* = .237^c^	Not included
Daily ASM load [related to DDD], median (IQR)	.7 (.6–1.0)	.7 (.6–1.0)	.7 (.6–1.0)	*p* = .762^c^	Not included
Prior ASM discontinuation attempt(s), *n* (%)	40 (20.4)	15 (17.4)	25 (22.7)	*p* = .362^b^	Not included
2‐year recurrence risk, %, median (IQR)		57.0 (46.0–70.0)	44.0 (27.0–57.0)	***p* < .001** ^c^	Not included
5‐year recurrence risk, %, median (IQR)		70.0 (57.0–81.0)	55.0 (35.0–70.0)	***p* < .001**.^c^	Not included

*Note*: Values which were statistically significant are marked as bold.

Abbreviations: ASM, antiseizure medication; CI, confidence interval; DDD, defined daily dose; IQR, interquartile range; OR, odds ratio.

^a^ For each patient, the neurologists' variables were considered.

^b^ Pearson chi‐squared test.

^c^ Mann–Whitney *U*‐test.

^d^ Due to multicollinearity with “duration of experience as neurologists” not included in logistic regression analysis.

^e^ Due to multicollinearity with “duration of seizure freedom” not included in logistic regression analysis.

^f^ Relationship status “single” compared to all other relationship statuses; structural epilepsy etiology compared to all other etiologies; total number of ≥10 seizures compared to total number of 1–9 seizures.

### 3.2 Shared decision‐making on discontinuation of ASM

Neurologists advocated for ASM discontinuation in 110 of 196 patients (56.1%), recommending complete discontinuation in 76 (69.1%) and a dose reduction of at least 25% in 34 (30.9%; Figures 1 and 2). Among these 110 patients, 29 (26.4%) chose to follow their neurologists' recommendation, whereas 81 opted for non‐discontinuation instead. Of the 76 patients to whom the neurologists recommended complete ASM discontinuation, seven (9.2%) decided to follow. A total of 22 patients decided to reduce their dose by at least 25%. Notably, in 11 of these 22 patients, their neurologist had recommended complete discontinuation, but the patients decided to reduce the dose instead (Figure 2).

### 3.3 Reasons for ASM non‐discontinuation

**FIGURE 3:**
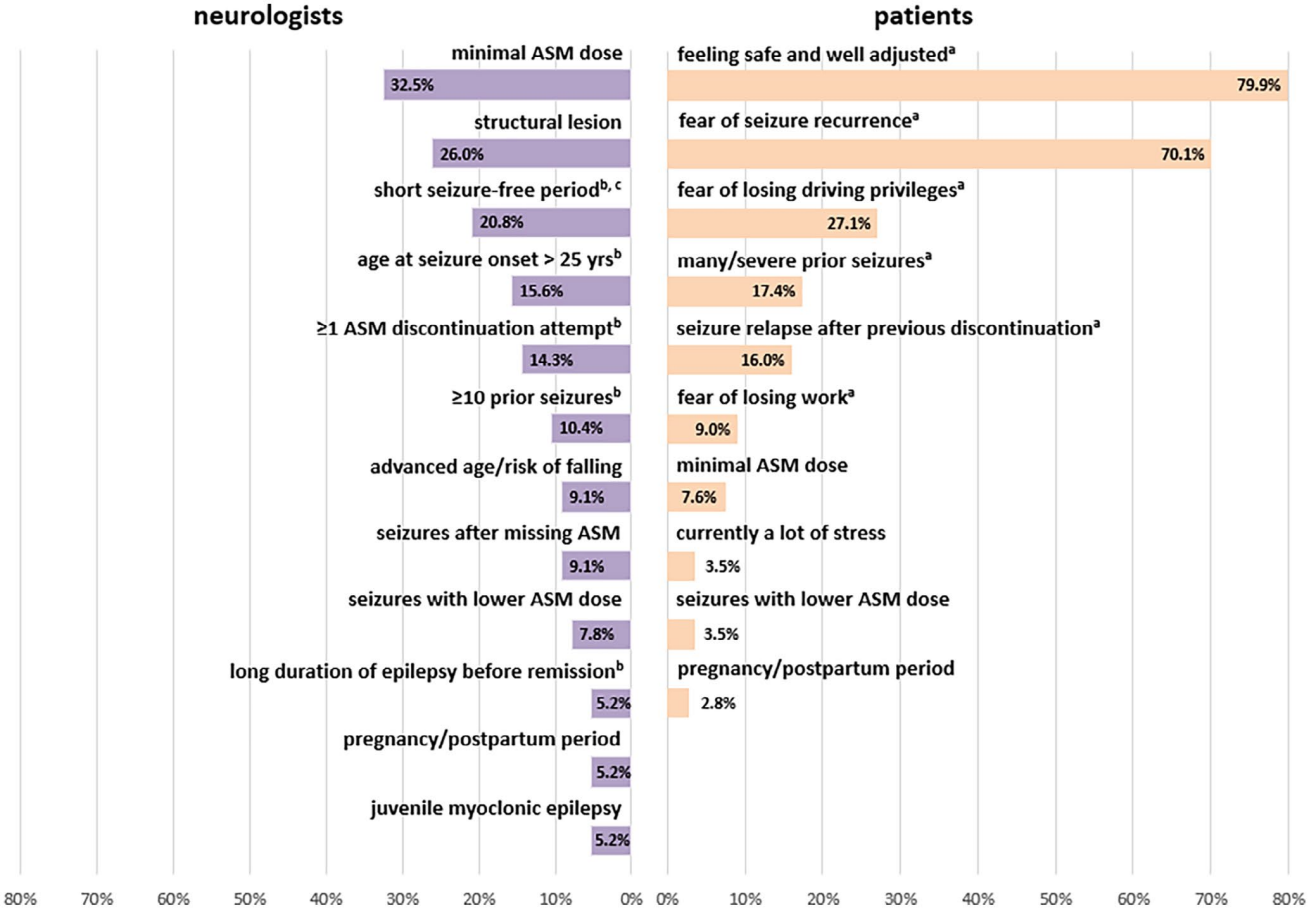
Reasons of neurologists and patients for non‐discontinuation of antiseizure medication (ASM). Multiple reasons were allowed for both groups. Neurologists provided reasons for not recommending ASM discontinuation in 86 patients, whereas 147 patients gave reasons for favoring non‐discontinuation. This figure indicates all reasons that were given by at least four respondents. For further reasons, see Table S1 for neurologists and Table S2 for patients. ^a^Predefined reasons based on a study examining patients' reasons for non‐discontinuation of ASM.^12^ ^b^Predefined reasons based on clinical variables independently associated with seizure recurrence after ASM discontinuation in a large meta‐analysis.^10^ ^c^Even though patients were seizure‐free for a minimum of 2 years, their neurologist still felt the seizure‐free period was too short for ASM discontinuation (mean seizure‐free period was 3 years).

Neurologists cited “patient already taking a minimal ASM dose” (32.5%) and “structural lesion” (26.0%) as most common reasons for ASM non‐discontinuation (Figure 3). Among all 196 patients surveyed, 147 favored non‐discontinuation. The most frequently given reasons were “feeling safe and well‐adjusted with ASM” (79.9%), “fear of seizure recurrence” (70.1%), and “fear of temporarily losing driving privileges” (27.1%; Figure 3). For reasons cited by ≤3 respondents, see Table S1 (neurologists) and Table S2 (patients). In the subgroup of 81 patients to whom neurologists recommended discontinuation but who opted for non‐discontinuation instead, these three reasons were also the most frequently cited (Table S3).

### 3.4 Variables associated with ASM non‐discontinuation in neurologists and patients

**FIGURE 4:**
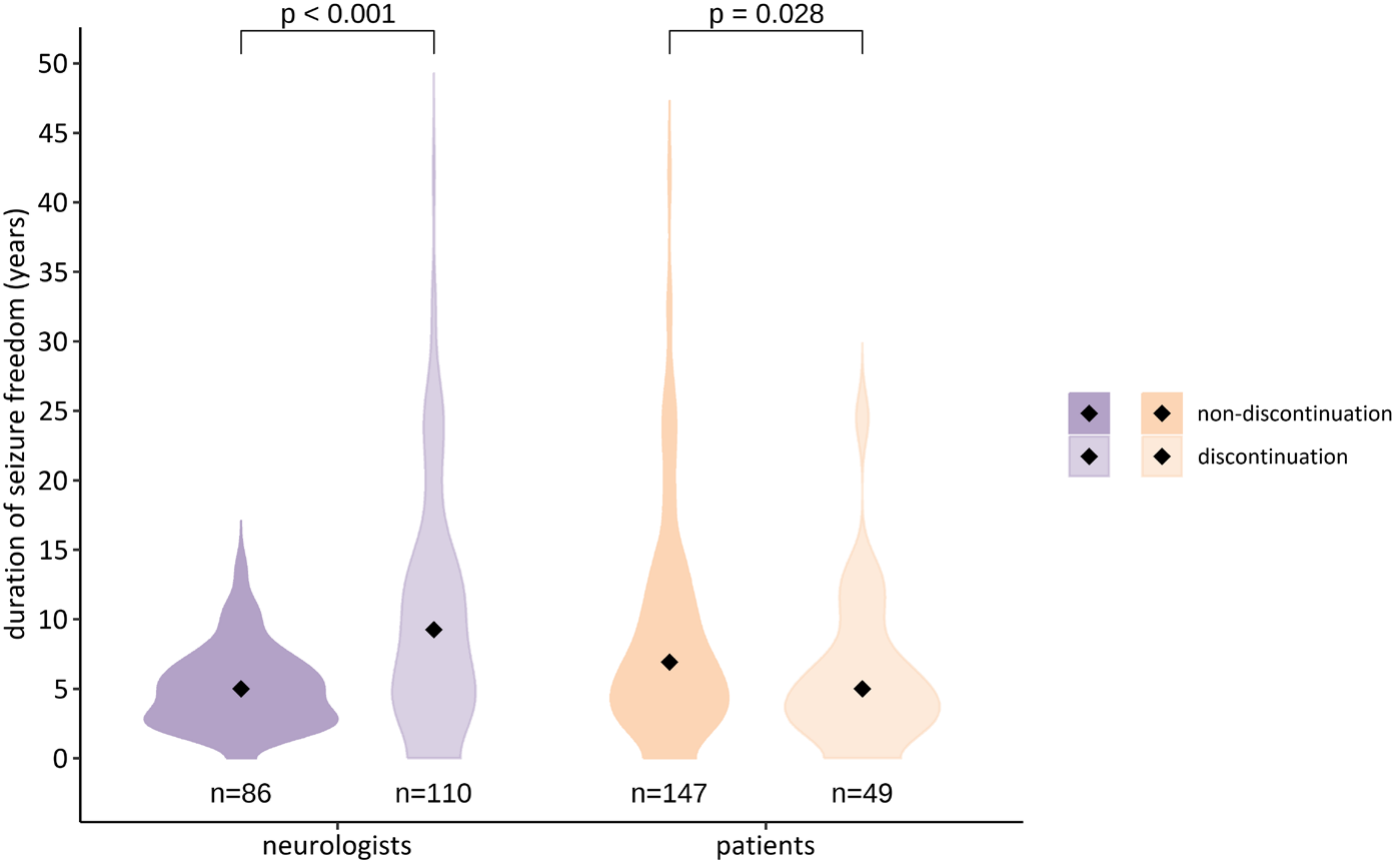
Duration of seizure freedom and association with antiseizure medication (ASM) non‐discontinuation and discontinuation. Violin plots show duration of seizure freedom prior to the shared decision‐making process between neurologists and patients on potential discontinuation of ASM. The black diamonds in the middle of the violins represent the median in each group. Duration of seizure freedom is significantly shorter in patients for whom neurologists opted for ASM non‐discontinuation (left side). In patients, duration of seizure freedom is significantly longer in those favoring ASM non‐discontinuation (right side).

Taking all 196 patients into account, predictors of ASM non‐discontinuation on the neurologists' side included fewer years of experience as neurologists (OR = .96, 95% CI = .92–.99) and a shorter duration of patients' seizure freedom (OR = .98, 95% CI = .98–.99; Table 1 and Figure 4). Excluding the 34 patients for whom the neurologists recommended dose reduction and only analyzing the subgroup of 76 patients for whom neurologists recommended complete discontinuation compared to the 86 patients who decided for non‐discontinuation, a shorter seizure‐free duration remained a predictor of non‐discontinuation (OR = .98, 95% CI = .98–.99; see Table S4).

Furthermore, in univariable analysis, the calculated seizure recurrence risk was significantly higher in patients for whom neurologists advocated ASM non‐discontinuation (2‐year risk: 57% vs. 44%, 5‐year risk: 70% vs. 55%; Table 1).

Next, we assessed all 196 patients' attitudes toward ASM discontinuation, regardless of their neurologists' recommendation. Among those, 147 (75.0%) opted for non‐discontinuation, 22 (11.2%) for complete discontinuation, and 27 (13.8%) for a dose reduction of at least 25% (Figure 1). Considering all patients, predictors of patients' preference for ASM non‐discontinuation included a history of generalized or focal to bilateral tonic–clonic seizures (OR = 2.72, 95% CI = 1.15–6.43) and a longer duration of seizure freedom (OR = 1.01, 95% CI = 1.01–1.02; Figure 4; for all findings, see Table 2). Excluding the 27 patients who favored a dose reduction and analyzing only the subgroup of 22 patients who opted for complete ASM discontinuation compared to the 147 patients who opted for non‐discontinuation, the latter remained independently associated with a longer duration of seizure freedom (OR = 1.02, 95% CI = 1.01–1.03). Further predictors comprised higher anxiety scores assessed by the GAD‐7 (OR = 1.37, 95% CI = 1.05–1.78) and better ASM tolerability (OR = 1.04, 95% CI = 1.01–1.07) as measured by the QOLIE‐31‐P (see Table S5).

**TABLE 2:** Clinical variables associated with preference of patients for non‐discontinuation of ASM.

Variable	Non‐discontinuation, *n* = 147	Discontinuation, *n* = 49	Univariable analysis	Logistic regression analysis, OR (95% CI)
Neurologists				
Neurologists advocating for discontinuation, *n* (%)	81 (55.1)	29 (59.2)	*p* = .618^a^	Not included
Patients: clinical variables				
Female sex, *n* (%)	81 (55.1)	23 (46.9)	*p* = .321^a^	Not included
Age, years, median (IQR)	52 (38–60)	43 (31–66)	*p* = .202^b^	Not included^c^
Relationship status “single”, *n* (%)	33 (22.4)	18 (36.7)	***p* = .048** ^a^	.54 (.24–1.20)^d^
Age at epilepsy onset, years, median (IQR)	20 (14–41)	22 (15–43)	*p* = .574^b^	1.01 (.99–1.03)
Duration of epilepsy until remission, years, median (IQR)	9 (2–20)	8 (1–18)	*p* = .383^b^	1.00 (.97–1.04)
Duration of seizure freedom, years, median (IQR)	7 (4–11)	5 (3–9)	***p* = .028** ^b^	**1.01 (1.01–1.02)**
Previous generalized or focal to bilateral tonic–clonic seizures, *n* (%)	128 (87.1)	35 (71.4)	***p* = .011** ^a^	**2.72 (1.15–6.43)**
Total number of seizures ≥10, *n* (%)	51 (34.7)	12 (25.5)	*p* = .185^a^	1.91 (.78–4.69)^d^
Epilepsy type, *n* (%)				
Focal	88 (59.9)	31 (63.2)	*p* = .442^a^	Not included
Generalized	39 (26.5)	9 (18.4)		
Unknown	20 (13.6)	9 (18.4)		
Structural epilepsy etiology, *n* (%)	40 (27.2)	12 (24.5)	*p* = .709^a^	Not included
Total number of ASMs [including current ASM], median (IQR)	2 (1–3)	1 (1–2)	*p* = .243^b^	Not included
Daily ASM load [related to DDD], median (IQR)	.7 (.6–1.1)	.7 (.5–.8)	***p* = .040** ^b^	2.37 (.98–5.71)
Prior discontinuation attempt(s), *n* (%)	31 (21.1)	9 (18.4)	*p* = .682^a^	Not included
2‐year recurrence risk, %, median (IQR)	52.0 (37.0–66.0)	52.0 (43.0–64.0)	*p* = .471^b^	Not included
5‐year recurrence risk, %, median (IQR)	64.0 (47.0–78.0)	64.0 (53.0–77.0)	*p* = .471^b^	Not included
Patients: questionnaires				
PESOS, median (IQR)				
Coping with epilepsy	24 (14–37)	24 (14–32)	*p* = .601^b^	Not included
Restrictions in daily living due to epilepsy	2 (0–10)	2 (0–10)	*p* = .636^b^	Not included
Felt stigma	0 (0–13)	0 (0–8)	*p* = .402^b^	Not included
Epilepsy‐related fear	21 (9–42)	21 (6–35)	*p* = .278^b^	Not included
LSSS, median (IQR)				
Percept subscale	26 (23–28)	26 (23–29)	*p* = .638^b^	Not included
Ictal/postictal subscale	29 (24–35)	30 (22–34)	*p* = .381^b^	Not included
LAEP, median (IQR)	31 (24–40)	30 (22–38)	*p* = .324^b^	Not included
QOLIE‐31‐P, median (IQR)	82 (74–88)	82 (75–90)	*p* = .574^b^	Not included
NDDI‐E, median (IQR)	9 (7–12)	10 (7–12)	*p* = .711^b^	Not included
GAD‐7, median (IQR)	3 (1–6)	3 (0–6)	*p* = .431^b^	Not included
BFI‐10, median (IQR)				
Extraversion	4 (3–5)	4 (3–4)	*p* = .180^b^	Not included
Neuroticism	3 (2–4)	3 (2–3)	*p* = .361^b^	Not included
Openness	4 (3–5)	4 (3–5)	*p* = .909^b^	Not included
Conscientiousness	4 (4–5)	4 (3–5)	*p* = .528^b^	Not included
Tolerance	4 (3–4)	4 (3–4)	*p* = .440^b^	Not included

*Note*: Values which were statistically significant are marked as bold.

Abbreviations: ASM, antiseizure medication; BFI‐10, Big Five Inventory; CI, confidence interval; DDD, defined daily dose; GAD‐7, Generalized Anxiety Disorder–7 items; IQR, interquartile range; LAEP, Liverpool Adverse Events Profile; LSSS, Liverpool Seizure Severity Scale; NDDI‐E, Neurological Disorders Depression Inventory for Epilepsy; OR, odds ratio; PESOS, Performance, Socio‐Demographic Aspects, Subjective Evaluation (questionnaire collecting information on the patients' social environment, coping with epilepsy, social impairment, and perceived stigma); QOLIE‐31‐P, Patient‐Weighted Quality of Life in Epilepsy.

^a^ Pearson chi‐squared test.

^b^ Mann–Whitney *U*‐test.

^c^ Due to multicollinearity with “duration of seizure freedom” not included in logistic regression analysis.

^d^ Relationship status “single” compared to all other relationship statuses; total number of ≥10 seizures compared to total number of 1–9 seizures.

A further subgroup analysis focused on the 110 patients for whom neurologists recommended ASM discontinuation. Here, logistic regression analysis also identified a longer duration of seizure freedom (OR = 1.01, 95% CI = 1.01–1.02) and, furthermore, a higher daily ASM load (OR = 8.18, 95% CI = 1.64–40.76) as predictors of ASM non‐discontinuation (see Table S6).

### 3.5 Follow‐up after ASM discontinuation

Of the 29 patients who decided to discontinue ASM following shared decision‐making with their neurologists, 28 (96.6%) proceeded with discontinuation as planned. Seven patients discontinued ASM completely as agreed, and 21 reduced the dose by at least 25%. One patient reduced the dose by only 15%. So far, after a median follow‐up of 7 months (IQR = 5–12), 25 of the 28 patients (89.3%) have remained seizure‐free. Three patients experienced seizure recurrence after a median of .5 months (range = .5–3 months) following the initiation of ASM dose reduction. None of these patients had completely discontinued ASM before seizures recurred. In two of the three cases, ASM was reintroduced to the previous dose, whereas in the third patient, ASM was resumed at a lower dose than before.

## 4 DISCUSSION

In seizure‐free patients with epilepsy, discontinuing ASM is a complex clinical decision that needs to involve careful assessment of risks and benefits as well as patients' individual preferences. Shared decision‐making is critical in this process, as it integrates clinical evidence with patients' attitudes to reach an individualized decision. This study provides deeper insights into the shared decision of neurologists and patients to discontinue ASM after some years of seizure freedom. Here, 85% of almost 200 patients who have been seizure‐free for a median of 6 years remained on their ASM and dose after the neurologists and the patients had considered and discussed the option of complete discontinuation or significant dose reduction.

We investigated reasons for neurologists to recommend and for patients to favor non‐discontinuation of ASM. The main reason given by the neurologists was that their respective patients were already taking the minimum ASM dose. In a prospective randomized study of 94 patients with epilepsy, 45 were assigned to complete ASM discontinuation and 49 remained on a low dose of ASM. In the survival analysis, the probability of remaining seizure‐free did not differ between the two groups (34% vs. 33%).^26^ Thus, taking a minimal ASM dose is rather an argument for and not against complete discontinuation. Interestingly, for patients a minimal ASM dose was only a subordinate reason for non‐discontinuation of ASM. Furthermore, in the subgroup of the 110 patients for whom the neurologists advocated discontinuation, a higher daily ASM load was independently associated with the decision for non‐discontinuation; this confirms what we have previously demonstrated in a retrospective study.^13^

The most frequent reasons specified by all surveyed patients for ASM non‐discontinuation were “feeling safe and well‐adjusted with their ASM” followed by “fear of recurrence of seizures” and “fear of temporarily losing driving privileges.” These motives are very plausible and were similar to those previously reported in two other studies investigating patients' attitudes on discontinuation of ASM.^12^, ^27^ For both neurologists and patients, non‐discontinuation was independently associated with duration of seizure freedom but in diametrically opposed directions. This also applied in the respective subgroup analyses where neurologists recommended and where patients favored complete ASM discontinuation. Neurologists recommended non‐discontinuation in patients with a shorter duration of seizure freedom. This is in line with a recent retrospective study in a cohort of adult patients and children that demonstrated that the probability of discussing discontinuation of ASM increased with a longer duration of seizure freedom.^14^ This approach is further supported by a meta‐analysis on seizure recurrence risk after ASM discontinuation; longer seizure freedom prior to stopping ASM was one of the major independent variables for remaining seizure‐free.^10^ Interestingly, in our study, the contrary was the case for patients; a longer duration of seizure freedom was independently associated with a higher likelihood for ASM non‐discontinuation. This was a robust predictor across all patients surveyed and in the subgroups analyzed. The association held true in the subgroup of patients who opted for complete discontinuation (excluding those who chose only a ≥25% dose reduction) compared to those who favored non‐discontinuation, as well as in the subgroup of 110 patients whose neurologists recommended discontinuation. A previous retrospective study discussed that longer duration of seizure freedom may serve as an argument for both discontinuation and non‐discontinuation of ASM.^14^ On the one hand, long‐term seizure freedom is a marker for a lower risk constellation, which may favor ASM discontinuation. However, on the other hand, for patients this may be an argument for non‐discontinuation due to the given seizure freedom, the lack of driving and occupational restrictions, and perceived therapeutic benefit from ASM.^14^

In the current study, a shorter duration of experience as neurologists was independently associated with non‐discontinuation of ASM. This is in line with the findings of a worldwide electronic survey on discontinuation of ASM surveying 466 physicians including neurophysiologists and epileptologists from 53 different countries. Physicians who were <10years in practice were less likely to taper ASM in a paradigmatic case with focal epilepsy of unknown etiology.^28^

In patients, a history of generalized or focal to bilateral tonic–clonic seizures was independently associated with non‐discontinuation of ASM. This association is intuitively comprehensible and confirms findings from a previous retrospective study from our center.^13^

Following univariable analysis, neurologists opted significantly more often for ASM non‐discontinuation if patients were living as single. Perhaps professionals regarded patients in a relationship as better supported and supervised if seizures reoccurred after ASM discontinuation, in particular with respect to sudden unexpected death in epilepsy, given the constellation that generalized or focal to bilateral tonic–clonic seizures in patients sleeping alone is associated with an almost 70‐fold increased risk.^29^ Interestingly, in our study, the opposite was the case for patients. They would opt for ASM non‐discontinuation significantly more often when living in a relationship, confirming previous data.^12^ At least in some cases, not only the patient but also the accompanying partner decides on discontinuation of ASM. In particular, generalized or focal to bilateral tonic–clonic seizures may lead to uncertainties in witnessing partners or other family members, which may in part explain the hesitance to discontinue ASM in patients living in a relationship.

In addition to clinical characteristics, our findings highlight the relevance of psychosocial factors in the decision‐making process on ASM discontinuation. In a subgroup analysis including patients who favored complete discontinuation of medication, higher anxiety levels (GAD‐7) and better reported ASM tolerability (QOLIE‐31‐P) were independently associated with ASM non‐discontinuation. This suggests that anxiety and treatment tolerability may be particularly influential in the more definitive decision to completely discontinue ASM. To date, only few studies have explored psychosocial factors influencing ASM discontinuation. Two previous studies have suggested that fear of seizure recurrence may increase patients' perceived vulnerability and act as a barrier to discontinuation.^12^, ^30^ Experiencing fewer adverse effects and thus better ASM tolerability is also a plausible factor for non‐discontinuation, as low tolerability of ASM is correlated with low quality of life^31^ and thus may result in self‐initiated ASM withdrawal.^32^ Taken together, these findings underscore that in the shared decision‐making process on ASM discontinuation, not only epilepsy‐related variables but also patients' emotional state and subjective treatment experiences need consideration. This is supported by findings from Terman et al., who showed that such concerns remained dominant even among patients with objectively low seizure relapse risk; highlighting that in patients' decision‐making, emotional and lifestyle‐related factors often outweigh statistical risk estimates.^16^

At our center, the “antiepileptic drug withdrawal risk calculator” was not routinely used by the neurologists or patients and was not part of the shared decision‐making process. However, for the purpose of this study, we subsequently calculated the seizure recurrence risks. Interestingly, in patients for whom neurologists advocated non‐discontinuation of ASM, the 2‐ and 5‐year calculated seizure recurrence risks were significantly higher. This reflects that the neurologists were well aware of the epilepsy‐related variables that are associated with an increased recurrence risk after ASM discontinuation.

Our study has strengths and limitations. We prospectively included quite a large number of 196 consecutive cases, and only 8% of 212 eligible patients declined participation or eventually were excluded. Patients were extensively phenotyped, and a plethora of standardized questionnaires were used to test for various psychosocial issues possibly associated with favoring non‐discontinuation of ASM. Patients were recruited from four different epilepsy outpatient clinics in Berlin (three academic belonging to one umbrella institution and one nonacademic), covering areas of the city with diverse socioeconomic backgrounds. This may allow for some generalization of the current findings, yet the regional concentration within a single metropolitan area in Germany limits the broader generalizability of results to other health care systems or geographic regions. All participating neurologists were from specialized tertiary epilepsy care settings, and their decision‐making may not reflect approaches in primary or general neurology care. Although 196 patients were included, the number still may be small when it comes to subgroup analyses, which may render some differences nonsignificant. Although seizure outcomes were not the primary objective of this study, we included a 6‐month follow‐up, that is, until the next outpatient visit, to obtain preliminary clinical outcome data after complete discontinuation or dose reduction. However, this short time frame and the low number of patients who eventually discontinued ASM are insufficient to make reliable and generalizable statements on seizure relapse risk. In our clinical setting, only a minority of patients routinely receive electroencephalographic (EEG) or brain magnetic resonance imaging scans prior to potential discontinuation of ASM. Thus, the potential impact of these two examinations could not be considered in our analyses. However, epileptiform EEG findings on seizure relapse risk after discontinuation seem to be overestimated by neurologists.^15^ Lastly, the observational and unblinded nature of the study may have introduced response or confirmation biases, as neurologists and patients were aware of the study's objectives. Nevertheless, efforts were made to standardize documentation and preserve open‐ended responses wherever possible.

## 5 CONCLUSIONS

Both neurologists and patients hesitate to discontinue ASM. In this study with long‐term seizure‐free patients, only 4% completely discontinued ASM and 11% reduced the dose. Whereas longer seizure freedom seems to motivate neurologists to recommend discontinuation, corresponding patients prefer non‐discontinuation. Despite concerns about ASM adverse effects, many patients fear seizure recurrence, especially those with a history of generalized or focal to bilateral tonic–clonic seizures. Health care providers should be aware of these diverging perspectives and engage patients in an open, empathetic shared decision‐making process regarding ASM discontinuation.

## AUTHOR CONTRIBUTIONS

**Maria Ilyas‐Feldmann:** Conceptualization; investigation; formal analysis; visualization; writing—original draft preparation. **Luise Graf:** Conceptualization; data collection; data curation; investigation; formal analysis; writing—review and editing. **Jakob I. Doerrfuss:** Investigation; formal analysis; visualization; writing—review and editing. **Matthias Dipper‐Wawra:** Investigation; visualization; writing—review and editing. **Nicoletta Doerr:** Investigation; writing—review and editing. **Rebekka Lehmann:** Investigation; writing—review and editing. **Christian Meisel:** Investigation; writing—review and editing. **David Steinbart:** Investigation; writing—review and editing. **Mirja Steinbrenner:** Investigation; writing—review and editing. **Martin Holtkamp:** Conceptualization; resources; supervision; writing—review and editing.

## FUNDING INFORMATION

This study was in part funded by the financial resources of the Friedrich von Bodelschwingh Endowed Professorship for Clinical and Experimental Epileptology at the Department of Neurology, Charité–Universitätsmedizin Berlin (to M.H.).

## CONFLICT OF INTEREST STATEMENT

M.I.‐F., L.G., M.D.‐W., N.D., R.L., C.M., D.S., and M.S. declare no conflicts of interest with respect to the research, authorship, and/or publication of this article. J.I.D. reports personal fees from Eisai within the past 3 years, outside the submitted work. M.H. reports personal fees from Angelini, Bial, Danone, Desitin, Eisai, Jazz Pharma, Neuraxpharm, and UCB. We confirm that we have read the Journal's position on issues involved in ethical publication and affirm that this report is consistent with those guidelines.

## Supporting information


Tables S1–S6.

## Data Availability

The data that support the findings of this study are available from the corresponding author upon reasonable request.
