# Supplementary material for: Non‐discontinuation of antiseizure medication in seizure‐free patients with epilepsy: Reasons and predictors among neurologists and patients
Source: Epilepsia. 2025 Jun 26;66(10):3785–98. doi: 10.1111/epi.18519 (PMC12605675; doi:10.1111/epi.18519)
Supplement: Supplementary file 1 — Tables S1–S6. [file EPI-66-3785-s001.docx]

**Supplementary Table 1 – Further reasons of neurologists recommending antiseizure medication non-discontinuation (n=86)**

|  | **n** |
| --- | --- |
| Psychiatric comorbidity | 3 |
| Upcoming exam | 3 |
| Prior breakthrough seizure | 3 |
| Good ASM compatibility | 3 |
| Recent ASM change | 1 |
| Narcolepsy | 1 |
| Missing information about MRI and  seizure recurrence(s) | 1 |
| Recent unclear paroxysmal events | 1 |
| Severe injuries during seizure(s) | 1 |
| Patient lives alone | 1 |
| Old generation ASM (reduction only  while in hospital) | 1 |
| EEG before withdrawal (reinsurance  for patient) | 1 |
| Leukoencephalopathy | 1 |
| Low ASM level | 1 |

N, number; ASM, antiseizure medication; reasons given by ≤3 respondents.

**Supplementary Table 2 – Further reasons of patients opting for antiseizure medication non-discontinuation (n=147)**

|  | **n** |
| --- | --- |
| Seizures after forgetting to take ASM | 2 |
| Recent ASM change | 2 |
| Getting a second opinion from  another doctor | 2 |
| Longer vacation (after that perhaps discontinuation attempt) | 1 |
| Impending fatherhood | 1 |
| Doctor advices to wait with  ASM discontinuation | 1 |
| Taking care of children | 1 |
| Severe injuries during seizures | 1 |
| Fear of refractory treatment after seizure recurrence(s) | 1 |
| Shift work | 1 |
| ASM helps with migraine | 1 |
| No additional insurance coverage without  taking ASM | 1 |
| Sleep disorder | 1 |
| Wish for further medical information | 1 |
| Desire to have children | 1 |
| Trusting doctor in choosing appropriate  ASM dosage | 1 |
| Finish college degree before reducing ASM | 1 |

N, number; ASM, antiseizure medication; reasons given by ≤3 respondents.

**Supplementary Table 3 – Reasons of patients to decide against neurologists’ recommendation for antiseizure medication discontinuation and to favor non-discontinuation (n=81)**

|  | **n** |
| --- | --- |
| Feeling safe and well adjusted | 62 |
| Fear of seizure recurrence | 60 |
| Fear of losing driving privileges | 23 |
| Seizure after discontinuation | 14 |
| Many/severe prior seizures | 14 |
| Fear of losing work | 8 |
| Minimal ASM dose | 5 |
| Currently a lot of stress | 3 |
| Seizure under lower ASM dose | 2 |
| Recent change of ASM | 2 |
| Getting a second opinion from another doctor | 2 |
| Longer vacation (after that perhaps discontinuation attempt) | 1 |
| Seizures after forgetting to take ASM | 1 |
| Pregnancy/Postpartum period | 1 |
| Planned pregnancy | 1 |
| Taking care of children | 1 |
| Fear of refractory treatment after seizure recurrence(s) | 1 |
| Shift work | 1 |
| ASM helps with migraine | 1 |
| Sleep disorder | 1 |
| Wish for further medical information | 1 |
| Finish college degree before reducing ASM | 1 |

N, number; ASM, antiseizure medication.

**Supplementary table 4 – Clinical variables associated with neurologists’ recommendation for antiseizure medication non-discontinuation in the subgroup of patients for whom neurologists recommended complete discontinuation compared to non-discontinuation**

|  | **Non-discontinuation (n=86)** | **Complete discontinuation (n=76)** | **Univariable analysis** | **Logistic regression analysis, OR (95%CI)** |
| --- | --- | --- | --- | --- |
| **Neurologists**^a^ |  |  |  |  |
| Female sex, n (%) | 31 (36.0) | 28 (36.8) | p= 0.916^b^ | Not included |
| Age, years, median (IQR) | 38 (34-52) | 41 (38-55) | **p= 0.005^c^** | Not included^d^ |
| Duration of experience as neurologists, years, median (IQR) | 11 (7-25) | 15 (11-27) | **p= 0.005^c^** | 0.96 (0.92–1.01) |
| **Patients**  Female sex, n (%) | 41 (47.7) | 43 (56.6) | p= 0.258^b^ | Not included |
| Age, years, median (IQR) | 46 (34-65) | 53 (39-62) | p= 0.167^c^ | Not included^e^ |
| Relationship status ‘single‘, n (%) | 30 (34.9) | 13 (17.1) | **p= 0.011^b^** | 2.03 (0.81–5.07)^f^ |
| Age at epilepsy onset, years, median (IQR) | 23 (16-47) | 19 (13-36) | p= 0.139^c^ | 1.01 (0.98–1.03) |
| Duration of epilepsy until remission,  years, median (IQR) | 10 (2-19) | 9 (1-20) | p= 0.793^c^ | 1.01 (0.97–1.05) |
| Duration of seizure freedom,  years, median (IQR) | 5 (3-7) | 11 (6-16) | **p< 0.001^c^** | **0.98 (0.97–0.99)** |
| Previous generalized or focal  to bilateral tonic-clonic seizures, n (%) | 74 (86.1) | 61 (80.3) | p= 0.324^c^ | Not included |
| Total number of seizures ≥ 10, n (%)  Epilepsy type, n (%) | 28 (32.6) | 26 (34.2) | p= 0.824^c^ | 1.05 (0.41–2.64)^f^ |
| Focal | 54 (62.8) | 43 (56.6) |  |  |
| Generalized | 22 (25.6) | 20 (26.3) | p= 0.571^b^ | Not included |
| Unknown | 10 (11.6) | 13 (17.1) |  |  |
| Structural epilepsy etiology, n (%) | 30 (34.9) | 13 (17.1) | **p= 0.011**^b^ | 1.48 (0.55–3.96)^f^ |
| Total number of ASM (including current ASM), median (IQR) | 1 (1-3) | 2 (1-3) | p= 0.627^c^ | Not included |
| Daily ASM load (related to DDD),  median (IQR) | 0.7 (0.6-1.0) | 0.7 (0.5-0.8) | p= 0.116^c^ | Not included |
| Prior ASM discontinuation attempt(s), n (%) | 15 (17.4) | 21 (27.6) | p= 0.120^b^ | Not included |
| 2-year recurrence risk, %, median (IQR) | 57.0 (46.0-70.0) | 42 (21-53) | **p< 0.001^c^** | Not included |
| 5-year recurrence risk, %, median (IQR) | 70.0 (57.0-81.0) | 53 (29-66) | **p< 0.001^c^** | Not included |

ASM, antiseizure medication; n, number; IQR, interquartile range; ^a^ For each patient, the neurologists’ variables were considered. ^b^ Pearson`s Chi-Squared-Test; ^c^ Mann-Whitney U Test; ^d^ due to multicollinearity with “duration of experience as neurologists” not included into logistic regression analysis; ^e^ due to multicollinearity with “duration of seizure freedom” not included into logistic regression analysis; ^f^ relationship status ‘single‘ compared to all other relationship status; structural epilepsy etiology compared to all other etiologies; total number of ≥ 10 seizures compared to total number of 1-9 seizures.

**Supplementary table 5 – Clinical variables associated with patients’ preference for antiseizure medication non-discontinuation compared to the subgroup of patients who decided for complete discontinuation**

|  | **Non-discontinuation (n=147)** | **Complete discontinuation**  **(n=22)** | **Univariable analysis** | **Logistic regression analysis, OR (95%CI)** |
| --- | --- | --- | --- | --- |
| **Neurologists** |  |  |  |  |
| Neurologists’ advocating for discontinuation, n (%) | 58 (39.5) | 7 (31.8) | p= 0.492^a^ | Not included |
| **Patients: Clinical variables** |  |  |  |  |
| Female sex, n (%) | 81 (55.1) | 9 (40.9) | p= 0.213^a^ | Not included |
| Age, years, median (IQR) | 52 (38-60) | 35 (26-54) | **p= 0.007^b^** | Not included^c^* |
| Relationship status ‘single‘, n (%) | 33 (22.4) | 9 (40.9) | p= 0.062^a^ | Not included^d^ |
| Age at epilepsy onset, years, median (IQR) | 20 (14-41) | 22 (11-44) | p= 0.953^b^ | 1.03 (0.99–1.07) |
| Duration of epilepsy until remission,  years, median (IQR) | 9 (2-20) | 3 (1-9) | **p= 0.017^b^** | 1.07 (0.99–1.15) |
| Duration of seizure freedom,  years, median (IQR) | 7 (4-11) | 4 (3-7) | **p= 0.007^b^** | **1.02 (1.01–1.03)** |
| Previous generalized or focal to bilateral tonic-clonic seizures, n (%) | 128 (87.1) | 13 (59.1) | **p< 0.001^a^** | 2.99 (0.69–12.98) |
| Total number of seizures ≥ 10, n (%) | 51 (34.7) | 6 (27.3) | p= 0.492^a^ | 0.86 (0.16–4.76)^d^ |
| Epilepsy type, n (%) |  |  |  |  |
| Focal | 88 (59.9) | 16 (72.2) | p= 0.202^a^ | Not included |
| Generalized | 39 (26.5) | 2 (9.1) |  |  |
| Unknown | 20 (13.6) | 4 (18.2) |  |  |
| Structural epilepsy etiology, n (%) | 40 (27.2) | 8 (36.4) | p= 0.375^a^ | Not included |
| Total number of ASM (including current ASM),  median (IQR) | 2 (1-3) | 1 (1-3) | p= 0.445^b^ | Not included |
| Daily ASM load (related to DDD), median (IQR) | 0.7 (0.6-1.1) | 0.7 (0.5-0.8) | p= 0.110^b^ | Not included |
| Prior discontinuation attempt(s), n (%) | 31 (21.1) | 3 (13.6) | p= 0.416^a^ | Not included |
| 2-year recurrence risk, %, median (IQR) | 52.0 (37.0-66.0) | 49.0 (40.0-60.0) | p= 0.718^b^ | Not included |
| 5-year recurrence risk, %, median (IQR) | 64.0 (47.0-78.0) | 60.0 (50.0-73.0) | p= 0.718^b^ | Not included |
| **Patients: Questionnaires** |  |  |  |  |
| **PESOS** |  |  |  |  |
| Coping with Epilepsy, median (IQR) | 24 (14-37) | 23 (15-32) | p= 0.719^b^ | Not included |
| Restrictions in daily living due to epilepsy,  median (IQR) | 2 (0-10) | 0 (0-10) | p= 0.188^b^ | Not included |
| Felt stigma, median (IQR) | 0 (0-13) | 0 (0-4) | p= 0.100^b^ | Not included |
| Epilepsy-related fear, median (IQR) | 21 (9-42) | 11 (0-22) | **p= 0.002^b^** | 1.05 (0.99–1.11) |
| **LSSS** |  |  |  |  |
| Percept subscale, median (IQR) | 26 (23-28) | 25 (21-29) | p= 0.564^b^ | Not included |
| Ictal/postictal subscale, median (IQR) | 29 (24-35) | 25 (19-30) | **p= 0.012^b^** | 1.03 (0.95–1.13) |
| **LAEP, median (IQR)** | 31 (24-40) | 29 (20-40) | p= 0.303^b^ | Not included |
| **QOLIE-31-P, median (IQR)** | 82 (74-88) | 87 (76-92) | p= 0.067^b^ | Not included |
| Medication side effects, median (IQR) | 100 (67-100) | 89 (51-100) | **p= 0.010^b^** | **1.04 (1.01–1.07)** |
| Seizure worry, median (IQR) | 83 (55-100) | 100 (79-100) | **p= 0.020^b^** | 0.99 (0.95–1.03) |
| **NDDI-E, median (IQR)** | 9 (7-12) | 9 (6-10) | p= 0.154^b^ | Not included |
| **GAD7, median (IQR)** | 3 (1-6) | 1 (0-3) | **p= 0.010^b^** | **1.37 (1.05–1.78)** |
| **BFI-10** |  |  |  |  |
| Extraversion, median (IQR) | 4 (3-5) | 4 (3-5) | p= 0.596^b^ | Not included |
| Neuroticism, median (IQR) | 3 (2-4) | 2 (1-4) | p= 0.106^b^ | Not included |
| Openness, median (IQR) | 4 (3-5) | 4 (3-5) | p= 0.772^b^ | Not included |
| Conscientiousness, median (IQR) | 4 (4-5) | 4 (2-5) | p= 0.364^b^ | Not included |
| Tolerance, median (IQR) | 4 (3-4) | 4 (3-4) | p= 0.187^b^ | Not included |

ASM, antiseizure medication; n, number; IQR, interquartile range; PESOS, questionnaire asking for the patients’ social environment, coping with epilepsy, social impairment and perceived stigma; LSSS, Liverpool Seizure Severity Scale; LAEP, Liverpool Adverse Events Profile; QOLIE-31-P, Patient-Weighted Quality of Life in Epilepsy; NDDI-E, Neurological Disorders Depression Inventory for Epilepsy; GAD7, Generalized Anxiety Disorder 7-item; BFI-10, Big Five Inventory; ^a^ Pearson`s Chi-Squared-Test; ^b^ Mann-Whitney U Test; ^c^ due to multicollinearity with “duration of seizure freedom” not included into logistic regression analysis; ^d^ relationship status ‘single‘ compared to all other relationship status; total number of ≥ 10 seizures compared to total number of 1-9 seizures.

**Supplementary table 6 – Clinical variables associated with patients’ preference for antiseizure medication non-discontinuation in subgroup of patients in whom neurologists recommended ASM discontinuation**

|  | **Non-discontinuation (n=81)** | **Discontinuation**  **(n=29)** | **Univariable analysis** | **Logistic regression analysis, OR (95%CI)** |
| --- | --- | --- | --- | --- |
| **Patients: Clinical variables** |  |  |  |  |
| Female sex, n (%) | 49 (60.5) | 14 (48.3) | p= 0.254^a^ | Not included |
| Age, years, mean (standard deviation) | 50 (15) | 54 (19) | p= 0.337^b^ | Not included^c^ |
| Relationship status ‘single‘, n (%) | 15 (18.5) | 6 (20.7) | p= 0.799^a^ | 0.39 (0.10–1.47)d |
| Age at epilepsy onset, years, median (IQR) | 17 (13-36) | 28 (19-44) | **p= 0.026^e^** | 0.98 (0.95–1.02) |
| Duration of epilepsy until remission,  years, median (IQR) | 9 (1-23) | 8 (1-21) | p= 0.809^e^ | 0.98 (0.94–1.03) |
| Duration of seizure freedom, years, median (IQR) | 10 (5-15) | 6 (4-12) | p= 0.055^e^ | **1.01 (1.01–1.02)** |
| Previous generalized or focal to bilateral tonic-clonic seizures, n (%) | 69 (85.2) | 20 (69.0) | p= 0.057^a^ | 1.52 (0.45–5.13) |
| Total number of seizures ≥ 10, n (%) | 26 (32.1) | 9 (31.0) | p= 0.916^a^ | 1.17 (0.35–3.91)^d^ |
| Epilepsy type, n (%) |  |  |  |  |
| Focal | 45 (55.6) | 20 (69.0) | p= 0.448^a^ | Not included |
| Generalized | 21 (25.9) | 5 (17.2) |  |  |
| Unknown | 15 (18.5) | 4 (13.8) |  |  |
| Structural epilepsy etiology, n (%) | 17 (21.0) | 5 (17.2) | p= 0.665^a^ | Not included |
| Total number of ASM (including current ASM),  median (IQR) | 2 (1-3) | 1 (1-3) | p= 0.301^e^ | Not included |
| Daily ASM load (related to DDD), median (IQR) | 0.8 (0.7-1.2) | 0.7 (0.5-0.7) | **p= 0.005^e^** | **8.18 (1.64–40.76)** |
| Prior discontinuation attempt(s), n (%) | 22 (27.2) | 3 (10.3) | p= 0.064^a^ | Not included |
| 2-year recurrence risk, %, mean (standard deviation) | 42.1 (21.5) | 48.5 (15.9) | p= 0.184^b^ | Not included |
| 5-year recurrence risk, %, mean (standard deviation) | 51.4 (24.0) | 59.3 (16.9) | p= 0.141^b^ | Not included |
| **Patients: Questionnaires** |  |  |  |  |
| **PESOS** |  |  |  |  |
| Coping with epilepsy, mean (standard deviation) | 26 (14) | 20 (11) | **p= 0.030^b^** | 1.04 (1.00–1.08) |
| Restrictions in daily living due to epilepsy,  median (IQR) | 2 (0-9) | 2 (0-10) | p= 0.672^e^ | Not included |
| Felt stigma, median (IQR) | 4 (0-10) | 0 (0-8) | p= 0.437^e^ | Not included |
| Epilepsy-related fear, median (IQR) | 24 (12-42) | 21 (8-38) | p= 0.278^e^ | Not included |
| **LSSS** |  |  |  |  |
| Percept subscale, median (IQR) | 26 (23-28) | 26 (24-28) | p= 0.670^e^ | Not included |
| Ictal/postictal subscale, mean (standard deviation) | 29 (8) | 28 (8) | p= 0.245^e^ | Not included |
| **LAEP, median (IQR)** | 29 (24-40) | 27 (21-37) | p= 0.300^e^ | Not included |
| **QOLIE-31-P, median (IQR)** | 85 (73-90) | 81 (74-90) | p= 0.981^e^ | Not included |
| **NDDI-E, median (IQR)** | 9 (7-12) | 10 (7-12) | p= 0.411^e^ | Not included |
| **GAD7, median (IQR)** | 3 (1-6) | 3 (1-6) | p= 0.766^e^ | Not included |
| **BFI-10** |  |  |  |  |
| Extraversion, median (IQR) | 4 (3-5) | 3 (3-4) | p= 0.175^e^ | Not included |
| Neuroticism, median (IQR) | 3 (2-4) | 3 (2-4) | p= 0.997^e^ | Not included |
| Openness, median (IQR) | 4 (3-5) | 4 (3-5) | p= 0.834^e^ | Not included |
| Conscientiousness, median (IQR) | 4 (4-5) | 4 (3-5) | p= 0.381^e^ | Not included |
| Tolerance, median (IQR) | 4 (3-4) | 4 (3-4) | p= 0.381^e^ | Not included |

ASM, antiseizure medication; n, number; IQR, interquartile range; PESOS, questionnaire asking for the patients’ social environment, coping with epilepsy, social impairment and perceived stigma; LSSS, Liverpool Seizure Severity Scale; LAEP, Liverpool Adverse Events Profile; QOLIE-31-P, Patient-Weighted Quality of Life in Epilepsy; NDDI-E, Neurological Disorders Depression Inventory for Epilepsy; GAD7, Generalized Anxiety Disorder 7-item; BFI-10, Big Five Inventory; ^a^ Pearson`s Chi-Squared-Test; ^b^ T-Test, ^c^ due to multicollinearity with “duration of seizure freedom” not included into logistic regression analysis; ^d^ relationship status ‘single‘ compared to all other relationship status; total number of ≥ 10 seizures compared to total number of 1-9 seizures.^e^ Mann-Whitney U Test
